# Stimulation of calcium-sensing receptors induces endothelium-dependent vasorelaxations via nitric oxide production and activation of IKCa channels

**DOI:** 10.1016/j.vph.2016.01.001

**Published:** 2016-05

**Authors:** Harry Z.E. Greenberg, Jian Shi, Kazi S. Jahan, Matthew C. Martinucci, Steven J. Gilbert, W.-S. Vanessa Ho, Anthony P. Albert

**Affiliations:** Vascular Biology Research Centre, Institute of Cardiovascular & Cell Sciences, St. George's, University of London, Cranmer Terrace, London SW17 0RE, UK

**Keywords:** Calcium-sensing receptors, Nitric oxide, BKCa, IKCa

## Abstract

Stimulation of vascular calcium-sensing receptors (CaSRs) is reported to induce both constrictions and relaxations. However, cellular mechanisms involved in these responses remain unclear. The present study investigates the effect of stimulating CaSRs on vascular contractility and focuses on the role of the endothelium, nitric oxide (NO) and K^+^ channels in these responses. In wire myography studies, increasing [Ca^2 +^]_o_ from 1 mM to 6 mM induced concentration-dependent relaxations of methoxamine pre-contracted rabbit mesenteric arteries. [Ca^2 +^]_o_-induced relaxations were dependent on a functional endothelium, and were inhibited by the negative allosteric CaSR modulator Calhex-231. [Ca^2 +^]_o_-induced relaxations were reduced by inhibitors of endothelial NO synthase, guanylate cyclase, and protein kinase G. CaSR activation also induced NO production in freshly isolated endothelial cells (ECs) in experiments using the fluorescent NO indicator DAF-FM. Pre-treatment with inhibitors of large (BKCa) and intermediate (IKCa) Ca^2 +^-activated K^+^ channels (iberiotoxin and charybdotoxin), and Kv7 channels (linopirdine) also reduced [Ca^2 +^]_o_-induced vasorelaxations. Increasing [Ca^2 +^]_o_ also activated IKCa currents in perforated-patch recordings of isolated mesenteric artery ECs. These findings indicate that stimulation of CaSRs induces endothelium-dependent vasorelaxations which are mediated by two separate pathways involving production of NO and activation of IKCa channels. NO stimulates PKG leading to BKCa activation in vascular smooth muscle cells, whereas IKCa activity contributes to endothelium-derived hyperpolarisations.

## Graphical abstract

**Figure:**
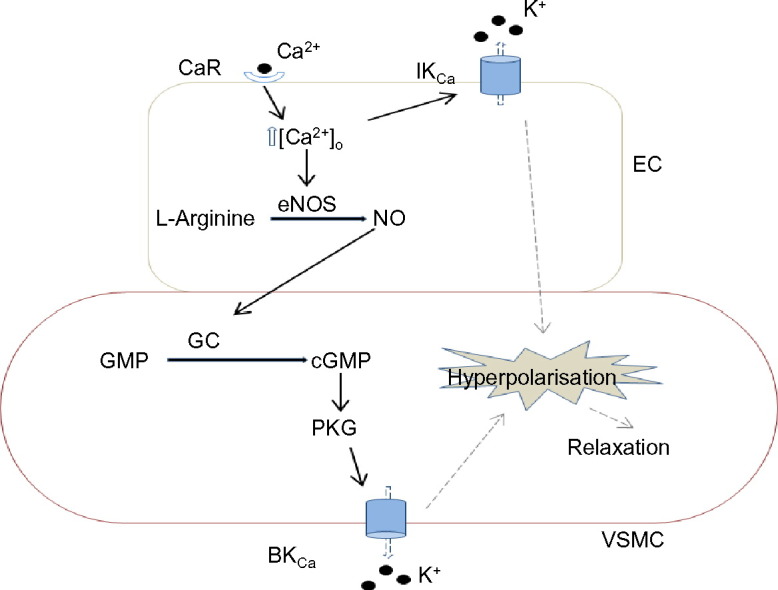

## 1 Introduction

Stimulation of plasmalemmal Ca^2 +^-sensing receptors (CaSRs) by an increase in the extracellular Ca^2 +^ concentration ([Ca^2 +^]_o_) is involved in maintaining plasma Ca^2 +^ homeostasis through regulation of parathyroid hormone from the parathyroid gland, intestinal Ca^2 +^ absorption, and renal Ca^2 +^ excretion [1]. It is also apparent that CaSRs are expressed in tissues not involved in plasma Ca^2 +^ homeostasis, including the Cardiovascular system [2]. In the presence of closely regulated plasma Ca^2 +^ levels, regulation of CaSRs is considered possible, as interstitial and localised [Ca^2 +^]_o_ is likely to vary sufficiently at the surface of cells [1], [3].

In the vasculature, CaSRs are expressed in perivascular nerves, endothelial cells (ECs), and vascular smooth muscle cells (VSMCs), and stimulation of these CaSRs is proposed to regulate vascular tone [2], [4]. Consequently, CaSRs and their associated cellular mechanisms are considered novel targets for controlling blood pressure. In addition, stimulation of CaSRs in the vasculature may also explain potential Cardiovascular risk following Ca^2 +^ supplements [5]. To date there is little consensus on the function of CaSRs in the vasculature, with findings suggesting that stimulation of CaSRs induces both vasoconstrictions and vasorelaxations through diverse cellular mechanisms [2], [4].

Stimulation of CaSRs on perivascular nerves is proposed to evoke synthesis and release of nitric oxide (NO) or vasoactive lipids from pre-synaptic terminals, which activate large conductance Ca^2 +^-activated K^+^ channels (BK_Ca_) in adjacent VSMCs that induces membrane hyperpolarisation and subsequent vasorelaxation [6], [7], [8], [9]. In addition, activation of CaSRs on ECs is reported to generate NO [10], [11], [12], [13] or induce activation of intermediate conductance Ca^2 +^-activated K^+^ channels (IK_Ca_) [14], [15], [16], which act as endothelium-derived relaxation or hyperpolarisation factors to also induce vasorelaxations. In contrast, CaSR-induced activation of Gq- and MAPK kinase-mediated pathways in VSMCs are linked to vasoconstriction [17], [18] and cell proliferation [19], [20], [21], [22] respectively. Taken together, these findings indicate that the effect of stimulating CaSRs in the vasculature is likely to be multifaceted.

However, many of the studies examining the functional role of vascular CaSRs have used calcimimetic compounds rather than endogenous CaSR ligands to activate the receptor. Importantly, these agents have also been shown to exhibit off-target actions within the vasculature for example by inhibiting voltage-gated Ca^2 +^ channels, by directly inducing endothelial nitric oxide generation, and by stimulating the opening of VSMC K^+^ channels [9], [11], thereby inducing vasorelaxations of pre-contracted vessels in a CaSR-independent manner. Accordingly, the mechanisms regulating vascular tone that are CaSR-mediated remain unclear. Moreover there is little information on the contribution of the proposed CaSR-mediated pathways in the same vascular preparation. Therefore the present work investigates CaSR-mediated effects induced by increasing [Ca^2 +^]_o_, an endogenous CaSR activator, and also focuses on the contribution of the endothelium, NO production and K^+^ channels in rabbit mesenteric arteries. In experiments using wire myography, fluorescence microscopy and electrophysiological techniques, we show that CaSRs are expressed in ECs and VSMCs, and that stimulation of CaSRs induces endothelium-dependent relaxations of methoxamine-induced pre-contracted vessels through production of NO and activation of IKCa channels.

## 2 Methods

### 2.1 Cell and vessel segment preparation

Male New Zealand White rabbits (2.5–3 kg) were killed by intravenous injection of sodium pentobarbitone (120 mgkg^− 1^) in accordance with Schedule I of the UK Animals Scientific Procedures Act, 1986. Second-order branches of rabbit superior mesenteric artery were dissected and cleaned of adherent tissue in physiological salt solution (PSS) containing (mM): NaCl 126, KCl 6, Glucose 10, HEPES 11, MgCl_2_ 1.2, and CaCl_2_ 1.5, with pH adjusted to 7.2 with 10 M NaOH. Following dissection, vessels were either cut into 2 mm segments for wire myography studies or were enzymatically dispersed to obtain freshly isolated ECs. To isolate single ECs, vessels were washed in PSS containing 50 μM [Ca^2 +^]_o_ for 5 min at 37 °C and placed in collagenase solution (1 mgmL^− 1^) for 15 min at 37 °C. The vessels were then triturated in fresh PSS and the cell-containing solution was collected and centrifuged for 1 min at 1000 rpm. The supernatant was removed and the cells re-suspended in fresh PSS containing 0.75 mM [Ca^2 +^]_o_, plated onto coverslips, and left at 4 °C for 1 h before use. To obtain isolated VSMCs, a similar technique was used except that initially the endothelium was gently removed from vessels with a cotton bud, and then the vessels were exposed to an enzyme solution containing collagenase (1 mgmL^− 1^) and protease (0.2 mgmL^− 1^) in 50 μM [Ca^2 +^]_o_ PSS for 15 min.

### 2.2 Isometric tension recordings

Effects of stimulating CaSRs on vascular tone were investigated using wire myography. Vessel segments of 2 mm in length were mounted in a wire myograph (Model 610 M; Danish Myo Technology, Aarhus, Denmark) and equilibrated for 30 min at 37 °C in 5 mL of gassed (95% O_2_/5% CO_2_) Krebs–Henseleit solution of the following composition (mM): NaCl 118, KCl 4.7, MgSO_4_ 1.2, KH_2_PO_4_ 1.2, NaHCO_3_ 25, CaCl_2_ 2, D-glucose 10. The mean resting diameter of the vessel segments was 422 ± 19 μm. Once mounted, vessel segments were then normalized to 90% of the internal circumference predicted to occur under a transmural pressure of 100 mmHg^23^. Mean vessel tensions were 5.3 ± 0.06 mN (n = 52, 253 vessel segments) in endothelium-intact vessels, and 5.2 ± 0.21 mN (n = 8, 18 vessel segments) in endothelium removed vessels after normalisation. After normalisation, vessels were left for 10 min and were then challenged with 60 mM KCl for 5 min. Endothelium integrity was then assessed by stably pre-contracting vessels with 10 μM methoxamine. Preliminary experiments demonstrated that 10 μM induced ~ 80% of the maximum methoxamine-induced contraction (data not shown). This was followed by the addition of 10 μM Carbachol (CCh) and vessels in which CCh-induced relaxations were > 90% of pre-contracted tone were designated as having a functional endothelium. When required, endothelium was removed by rubbing the intima with a human hair, and CCh-induced relaxations of < 10% of pre-contracted tone indicated successful removal. Vessel segments were incubated for 30 min in fresh Krebs solution containing 1 mM CaCl_2_ and then pre-contracted with 10 μM methoxamine, 300 nM U46619, or 60 mM KCl as required. This was followed by cumulative additions of CaCl_2_, increasing [Ca^2 +^]_o_ from 1 to 6 mM in the presence of inhibitors tested or their respective vehicles. All inhibitors were added to the vessel segments 30 min before the construction of concentration–response curves to [Ca^2 +^]_o_. For each experiment, vehicle controls were performed using vessel segments from the same animal. All relaxant responses are expressed as percentage relaxation of tension induced by either 10 μM methoxamine, 300 nM U46619, or 60 mM KCl. Data points on all graphs are mean values and error bars represent S.E.M. For each experiment n = number of animals, with at least 3–4 vessel segments used from each animal. Responses to increasing [Ca^2 +^]_o_ were analysed by 2-way ANOVA followed by Bonferroni post hoc tests. p < 0.05 was taken as statistically significant. Bonferroni comparisons are shown above the graph data points whereby: *p < 0.05, **p < 0.01, ***p < 0.001, ****p < 0.0001 vs. controls. EC_50_ and E_max_ values were also calculated and compared with Student's t-test. Statistical analysis and graphs were made using Graphpad Prism 6 software (GraphPad Software, Inc., San Diego, CA, USA).

### 2.3 Immunocytochemistry

Freshly dispersed ECs/VSMCs were fixed onto borosilicate coverslips with 4% paraformaldehyde (Sigma-Aldrich, Gillingham, UK) for 10 min, washed 4 times with phosphate-buffered saline (PBS), and permeabilised with PBS containing 0.1% Triton X-100 for 20 min at room temperature. Cells were then washed 4 times with PBS and incubated with PBS containing 1% bovine serum albumin (BSA) for 1 h at room temperature. The cells were then incubated overnight at 4 °C with mouse anti-CaSR antibody (1:200) and either goat anti-PECAM-1 (1:200) for ECs or goat anti-α-actin (1:100) for VSMCs in PBS containing 1% BSA. In control experiments, cells were incubated without primary antibodies. The cells were then washed 4 times with PBS and incubated with secondary antibodies conjugated to a fluorescent probe for 1 h (Alexa Fluor 546-conjugated donkey anti-mouse antibody (1:500) and Alexa Fluor 488-conjugated donkey anti-goat antibody (1:500). Unbound secondary antibodies were removed by washing with PBS, and nuclei were labelled with 4′,6-diamidino-2-phenylindole (DAPI) mounting medium (Sigma-Aldrich). Cells were imaged using a Zeiss LSM 510 laser scanning confocal microscope (CaSRl Zeiss, Jena, Germany). Excitation was produced by 543 nm or 488 nm lasers and delivered to the specimen via a Zeiss Apochromat × 63 oil-immersion objective. Emitted fluorescence was captured using LSM 510 software (release 3.2; CaSRl Zeiss). Two-dimensional images cut horizontally through the middle of the cells were captured and raw confocal imaging data processed using Zeiss LSM 510 software. Final images were produced using PowerPoint (Microsoft XP; Microsoft, Richmond, WA, USA).

### 2.4 Nitric oxide imaging

ECs were placed in a sterilised 96-well plate and left for 1 h at 4 °C. Cells were loaded with the nitric oxide fluorescent dye DAF-FM diacetate (1 μM), incubated at 4 °C for 20 min and then washed with PSS containing 1 mM [Ca^2 +^]_o_. The cells were then left for another 30 min at 4 °C to allow complete de-esterification of intracellular diacetate. Inhibitors tested or their respective vehicles were also added at this point. Changes in fluorescence following 5 min of CaSR stimulation with 6 mM [Ca^2 +^]_o_ were captured using a Zeiss Axiovert 200 M Inverted microscope and processed and analysed using AxioVision SE64 Software (Rel. 4.9.1; CaSRl Zeiss). 10 μM CCh was used as a positive control. Additional experiments were CaSRried out to investigate the origins of the baseline fluorescence observed in ECs loaded with DAF-FM diacetate. In these studies, fluorescence changes were recorded 30 min after the addition of the inhibitors tested or their respective vehicles. Changes in fluorescence were quantified by selecting a cell as a region of interest (ROI) and comparing fluorescence levels within the ROI before and after the experimental protocols and analysed using unpaired Student's t-test with p < 0.05 considered significant. Figures and analysis were made using Graphpad Prism 6 (GraphPad Software, Inc., San Diego, CA, USA).

### 2.5 Electrophysiology

Whole-cell and perforated-patch configurations of the patch clamp technique were used to record K^+^ conductances. Recordings were made with an Axopatch 200B amplifier (Axon Instruments, Union City, CA, USA) at room temperature (20–23 °C). Currents were filtered at 1 kHz (− 3 dB, low-pass 8-pole Bessel filter, Frequency Devices model LP02; Scensys, Aylesbury, UK) and sampled at 5 kHz (Digidata 1322 A and pCLAMP 9.0 software; Molecular Devices, Sunnydale, CA, USA). Currents were evoked by either dialysing cells with a pipette solution containing 3 μM free Ca^2 +^ or by bath applying 6 mM [Ca^2 +^]_o_ in whole-cell or perforated-patch recordings, respectively. Additional whole-cell recordings were made with a patch pipette solution containing no added Ca^2 +^. Current/voltage relationships (I/V) were obtained by applying a 200 ms voltage ramp from − 100 mV to + 100 mV every 30 s from a holding potential of − 60 mV. IK_Ca_ currents were calculated from the decrease in current produced by charybdotoxin (CbTx) and the presence of SK_Ca_ currents was calculated by any further decrease in current induced by co-application of apamin. I/V relationship are from mean data ± S.E.M., with each point from n = 6 patches and at least 3 animals. Data were analyzed using unpaired Student's t-test with p < 0.05 considered significant. Figures and analysis were made using MicroCal Origin 6.0 software (MicroCal Software, Northampton, MA, USA). The external bathing solution for both whole-cell and perforated-patch recordings contained as previously used [24] (mM): NaCl 134, KCl 6, Glucose 10, HEPES 10, MgCl_2_ 1, CaCl_2_ 1 (adjusted to pH 7.4 with 10 M NaOH). For whole-cell recordings, the pipette solution contained (mM): KCl 134, HEDTA 5, HEPES 10, MgCl_2_ 5.53 (1 mM free Mg^2 +^) and CaCl_2_ 0.207 (3 μM free Ca^2 +^) (pH 7.2). The amounts of MgCl_2_ and CaCl_2_ added were determined using EqCal software (Biosoft, Cambridge, UK). For experiments using a pipette solution containing no added Ca^2 +^, CaCl_2_ was omitted. For perforated-patch recordings the pipette solution contained (mM): K-aspartate 110, KCl 30, NaCl 10, HEPES 10, MgCl_2_ 1, pH 7.2 with 10 M NaOH, and amphotericin (200 μgmL^− 1^).

### 2.6 Materials

Mouse anti-CaSR (SC-47741), goat anti-PECAM-1 (SC-1506), goat anti-α-actin (SC-1616), and secondary antibodies were obtained from Santa Cruz Biotechnology (Dallas, TX, USA). All other materials were purchased from Sigma-Aldrich (Sigma Chemical Co., Poole, UK) or Tocris (Tocris Biosciences, Bristol, UK). Drugs were dissolved in distilled water or dimethyl sulfoxide (DMSO).

## 3 Results

### 3.1 Stimulation of CaSRs induces endothelium-dependent vasorelaxation of rabbit mesenteric arteries

**Fig. 1:**
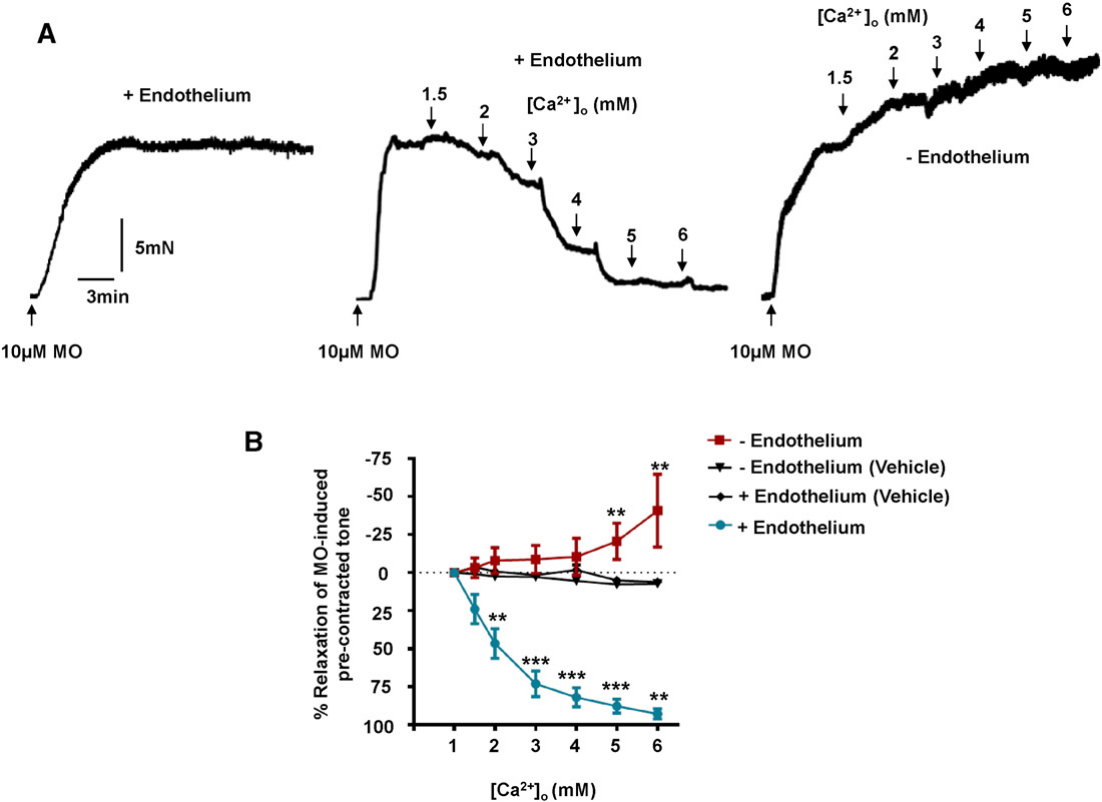
Effect of [Ca^2 +^]_o_ on pre-contracted tone of rabbit mesenteric arteries. (A) Representative traces showing the effect of [Ca^2 +^]_o_ on pre-contracted tone induced by 10 μM methoxamine (MO). The left record shows that MO induces a stable vasoconstriction in the presence of 1 mM [Ca^2 +^]_o_, whilst the middle and right records show the effect of increasing [Ca^2 +^]_o_ from 1 mM to 6 mM on endothelium-intact and endothelium-removed vessels respectively. (B) Mean data showing the effect of [Ca^2 +^]_o_ on MO-induced pre-contraction tone. Each point is from n = 8 animals, with at least n = 3 vessel segments per animal. **p < 0.01, ***p < 0.001.

In our initial experiments, we investigated the functional role of CaSRs in rabbit mesenteric arteries by studying the effect of increasing [Ca^2 +^]_o_ on pre-contracted tone evoked by 10 μM methoxamine. Fig. 1A shows that increasing [Ca^2 +^]_o_ from 1 mM to 6 mM induced concentration-dependent relaxations of rabbit mesenteric artery vessel segments compared to time-matched vehicle controls. Fig. 1A and B reveal that almost complete relaxation of pre-contracted tone was achieved with 6 mM [Ca^2 +^]_o_, and that physiological plasma [Ca^2 +^]_o_ levels (1–2 mM) relaxed pre-contracted tone between 30 and 50%. (EC_50_ ~ 2.2 mM [Ca^2 +^]_o_ and E_max_ ~ 96%, Table 1).

We next studied if removal of a functional endothelium had an effect on [Ca^2 +^]_o_-induced relaxations. Fig. 1A demonstrates that removal of a functional endothelium, determined by the absence of CCh-evoked relaxations of pre-contracted tone (see Methods), completely abolished [Ca^2 +^]_o_-induced relaxations. Instead, Fig. 1A and B reveal that in the absence of a functional endothelium increasing [Ca^2 +^]_o_ between 1 mM and 6 mM produced a concentration-dependent potentiation of pre-contracted tone, with 6 mM [Ca^2 +^]_o_ increasing contractility by over 50%. There was no difference in the mean amplitude of pre-contracted tone induced by 10 μM methoxamine between vessel segments with functional endothelium-intact compared to those with functional endothelium-removed (6.5 ± 0.6 mN, n = 8 vs 9.1 ± 2.1 mN respectively, n = 8 animals with 3–4 vessel segments from each animal, p > 0.05). These data indicate that stimulation of CaSRs exerts vasorelaxant and vasoconstrictor actions via endothelium- and vascular smooth muscle-dependent processes, respectively. In a separate experiment, the same phenomenon was demonstrated using a different vasoconstrictor agent to pre-contract the vessels, namely the thromboxane receptor agonist U46619, with increases in [Ca^2 +^]_o_ increases in [Ca^2 +^]_o_ evoking concentration-dependent vasorelaxations that were abolished with the removal of the endothelium (Supplementary Figure S1).

**Fig. 2:**
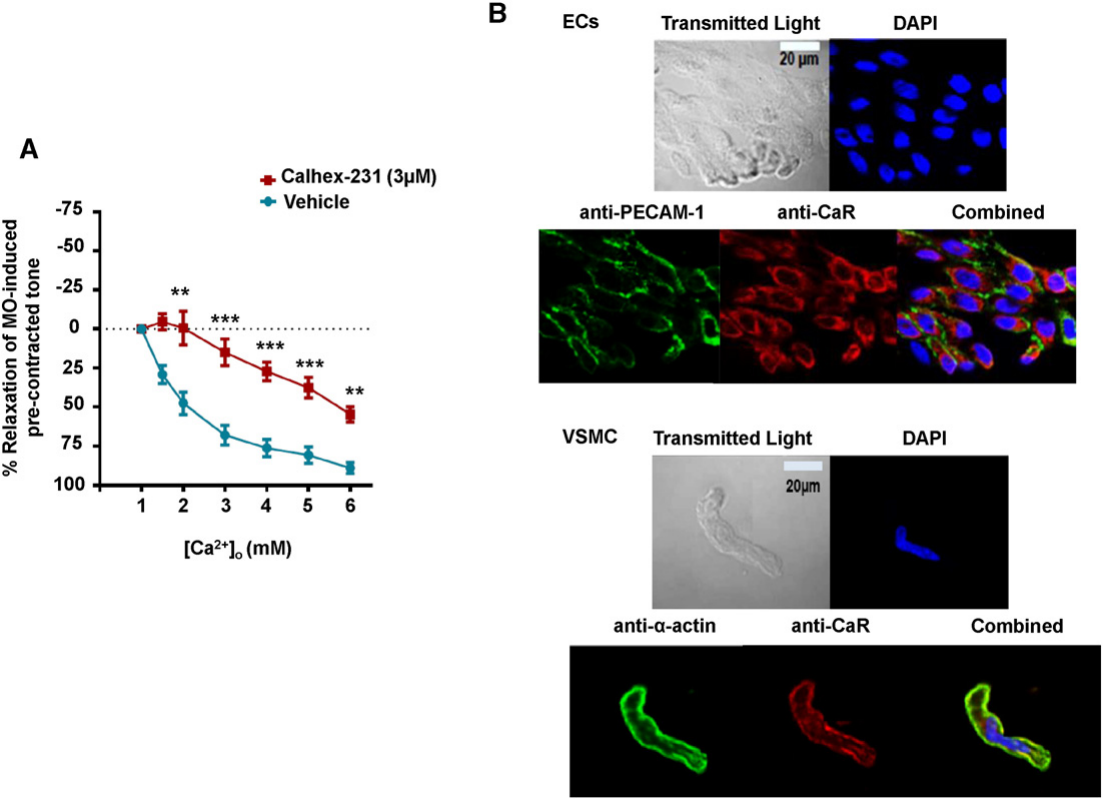
Relaxant effect of [Ca^2 +^]_o_ on pre-contracted tone involve CaSRs. (A) Mean data showing that relaxations of pre-contracted tone induced by increasing [Ca^2 +^]_o_ from 1 mM to 6 mM are reduced by pre-treatment with 3 μM Calhex-231. Each point is from n = 4 animals, with at least n = 3 segments per animal. **p < 0.01, ***p < 0.001. (B) Immunocytochemistry images showing that CaSRs are expressed in ECs and VSMCs. PECAM-1 and α-actin are selective markers for ECs and VSMCs respectively. DAPI stains the cell nucleus.

To examine if [Ca^2 +^]_o_-induced relaxations of methoxamine pre-contracted tone were mediated by stimulation of CaSRs, we pre-treated vessel segments with Calhex-231, a negative allosteric modulator of CaSRs [25]. Fig. 2A shows that 3 μM Calhex-231 inhibited [Ca^2 +^]_o_-induced relaxations compared to time-matched vehicle controls. In control experiments, 3 μM Calhex-231 had no effect on CCh-induced relaxations of pre-contracted tone (Supplementary Figure S2) indicating that this calcilytic did not affect the general ability of vessel segments to relax. Consistent with our ideas for an involvement of CaSRs, Fig. 2B shows that CaSRs were expressed in freshly isolated rabbit mesenteric ECs and VSMCs using immunocytochemistry.

These results provide clear evidence that stimulation of CaSRs induces a profound endothelium-dependent relaxation.

### 3.2 Generation of NO in endothelial cells contributes to CaSR-mediated relaxations

**Fig. 3:**
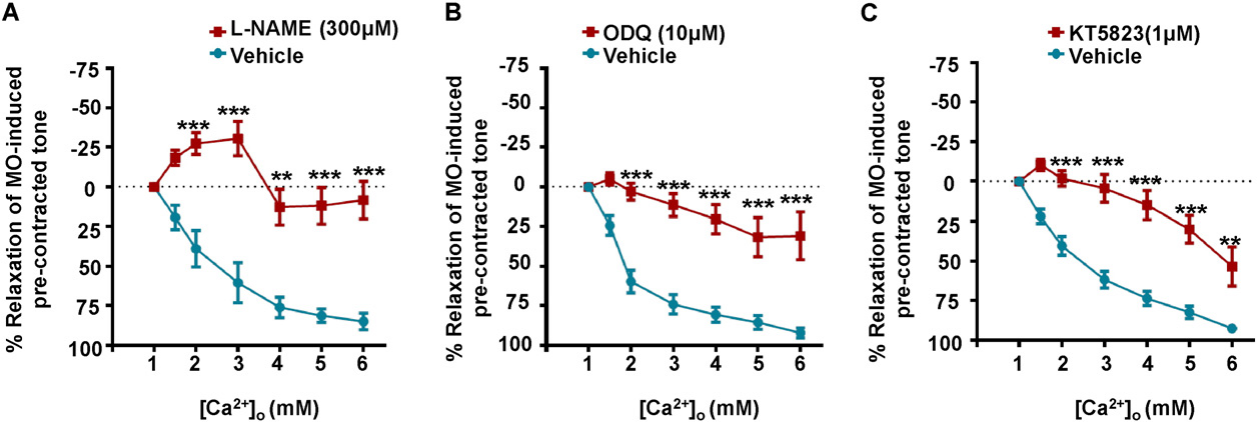
Effect of NO–GC–PKG pathway inhibitors on [Ca^2 +^]_o_-induced relaxations of pre-contracted tone. [Ca^2 +^]_o_-induced relaxations of pre-contracted tone were markedly inhibited by (A) the eNOS inhibitor L-NAME (300 μM), (B) the guanylate cyclase inhibitor ODQ (10 μM) and (C) the PKG inhibitor KT5823 (1 μM). Each point is from n = 4 animals, with at least n = 3 segments per animal. **p < 0.01, ***p < 0.001.

A role for NO has been proposed to mediate actions of CaSRs in the vasculature [10], [11], [12], and therefore we wished to explore if NO contributed to the CaSR-induced endothelium-dependent vasorelaxations described above. We first studied the effect of L-NAME, an endothelial NO synthase (eNOS) inhibitor, on [Ca^2 +^]_o_-induced relaxations of pre-contracted tone. Fig. 3A shows that pre-treatment with 300 μM L-NAME produced a pronounced inhibition of [Ca^2 +^]_o_-induced vasorelaxations. It is well-established that NO induces vasorelaxations through a pathway involving guanylate cyclase (GC), production of cGMP, and stimulation of protein kinase G (PKG) activity, which subsequently regulates contractile mechanisms in vascular smooth muscle [26]. Fig. 3B and C show that pre-treatment with the GC inhibitor, ODQ (10 μM), and the PKG inhibitor KT5823 (1 μM), also produced marked inhibition of [Ca^2 +^]_o_-induced relaxations of pre-contracted tone. Previous studies have shown that PKG may act to suppress EDH mechanisms and that inhibition with KT5823 can evoke significant EDH-mediated vasorelaxations [27]. As Supplementary Figure S3 shows, 1 μM KT5823 induced endothelium-dependent relaxations of pre-contracted tone which were inhibited by co-application of the IKCa blocker charybdotoxin (CbTX) and the SKCa blocker apamin. It is therefore possible that KT5823-mediated vasorelaxations may have contributed to an underestimation of the inhibitory action of KT5823 on CaSR-mediated responses.

**Fig. 4:**
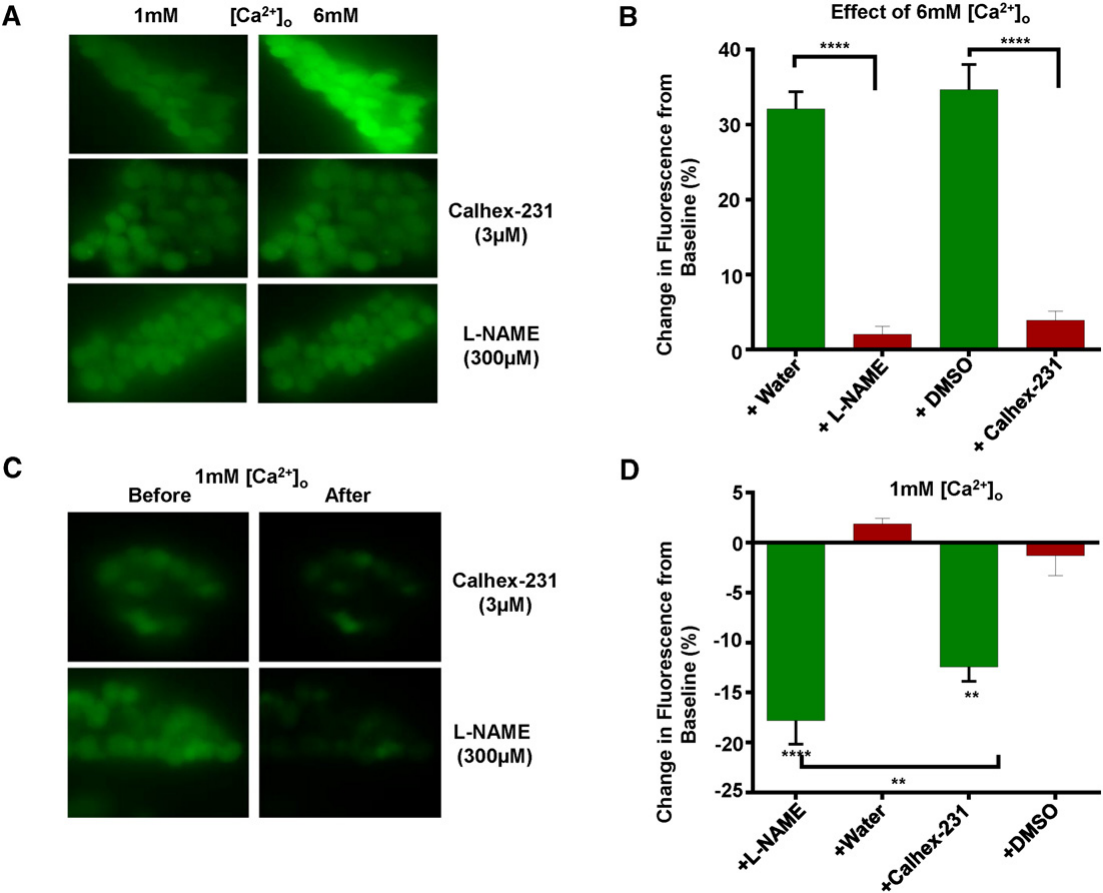
Effects of [Ca^2 +^]_o_ on NO production in ECs measured using DAF-FM fluorescence. (A) and (B) show original images and mean data showing that increasing [Ca^2 +^]_o_ from 1 mM to 6 mM enhanced basal fluorescence which was inhibited by pre-treatment with 3 μM Calhex-231 or 300 μM L-NAME. (C) and (D), representative images and mean data illustrating that basal fluorescence in 1 mM [Ca^2 +^]_o_ was inhibited by Calhex-231 and L-NAME. n = at least 50 cells from n = 4 animals. ** p < 0.01, ***p < 0.001.

To further investigate if stimulation of CaSRs evokes an increase in NO production, we studied the effect of increasing [Ca^2 +^]_o_ on NO generation in freshly isolated mesenteric artery ECs using the cell-permeable fluorescent NO indicator DAF-FM. Fig. 4A and B demonstrates that increasing [Ca^2 +^]_o_ from 1 mM to 6 mM enhanced basal fluorescence by over 30% compared to time-matched vehicle controls, and that these responses were reduced by pre-treatment with 3 μM Calhex-231 and 300 μM L-NAME. Fig. 4C and D also shows that basal fluorescent levels recorded in 1 mM [Ca^2 +^]_o_ were reduced by 3 μM Calhex-231 and 300 μM L-NAME. Positive control experiments revealed that CCh-evoked increases in fluorescence in 1 mM [Ca^2 +^]_o_ which were unaffected by 3 μM Calhex-231 but as has been shown previously [28], were inhibited by 300 μM L-NAME (Supplementary Figure S4).

These results provide strong evidence that stimulation of CaSRs evokes NO production in mesenteric artery ECs leading to PKG-dependent vasorelaxations.

### 3.3 Voltage-gated and Ca^2 +^-activated K^+^ channels also contribute to CaSR-mediated vasorelaxations

It is well recognised that IKCa and SKCa channels expressed in ECs have pivotal roles in mediating endothelium-derived hyperpolarisations which couple to relaxation of VSMCs [29], [30], and IKCa channel activity is also linked to CaSR-mediated hyperpolarisations and vasorelaxations [14], [15], [16]. It is also possible that other K^+^ channels such as Kv7 and K_ATP_ channels expressed in VSMCs may be involved in mediating CaSR-induced vasorelaxations [31], [32]. Therefore we examined the effect of BKCa, IKCa, SKCa, Kv7, and K_ATP_ channel inhibitors on [Ca^2 +^]_o_-induced relaxations of pre-contracted tone.

**Fig. 5:**
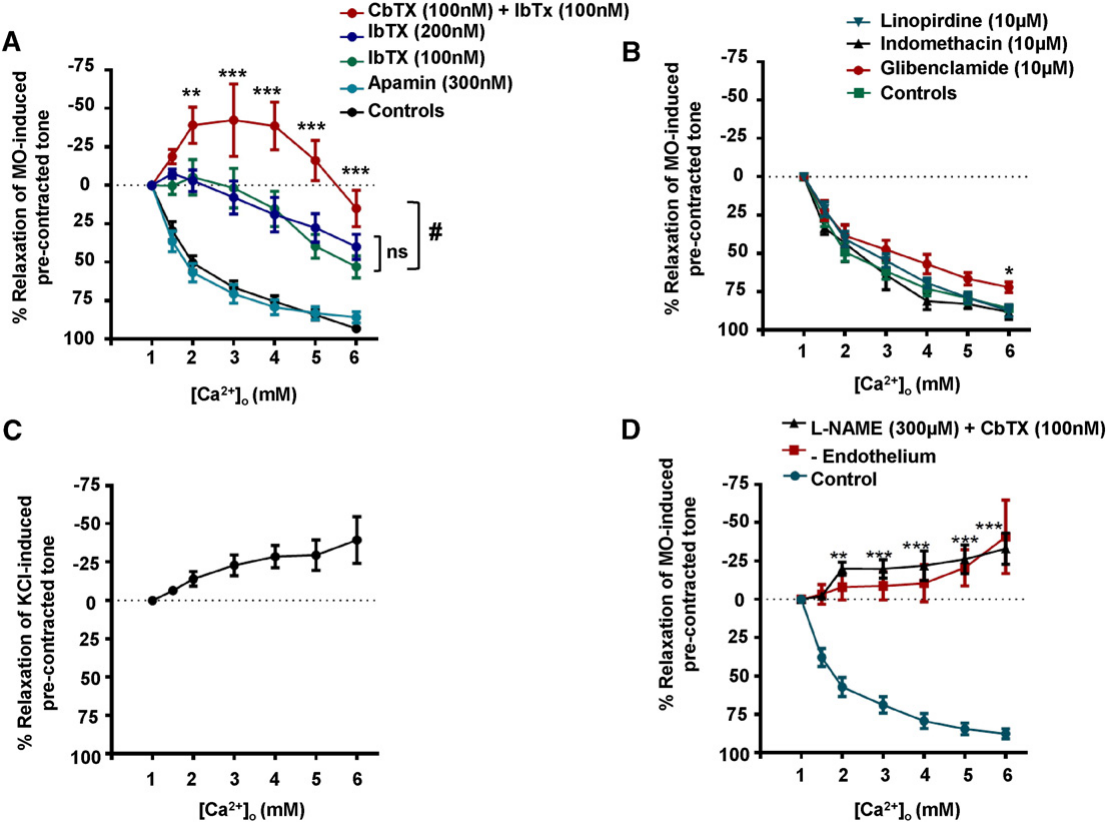
Effects of K^+^ channel blockers on [Ca^2 +^]_o_-induced relaxations of pre-contracted tone. The effects of pre-treating vessel segments with (A) IbTX (100 nM or 200 nM), apamin (100 nM), and CbTX together with IbTX (both 100 nM) and (B) linopirdine (10 μM), indomethacin (10 μM), or glibenclamide (10 μM) on [Ca^2 +^]_o_-induced relaxations of pre-contracted tone. (C) Effect of [Ca^2 +^]_o_ on 60 mM KCl-induced pre-contracted tone in endothelium-intact vessel segments. (D) Effect of L-NAME (300 μM) and CbTX (100 nM) on [Ca^2 +^]_o_-induced relaxations of pre-contracted tone. The effect of [Ca^2 +^]_o_ on endothelium-removed vessel segments (Fig. 1A) is also shown for comparison. #p < 0.05, *p < 0.05, **p < 0.01, ***p < 0.001.

Fig. 5A shows that pre-treatment with 100 nM iberiotoxin (IbTX), a selective BKCa inhibitor, markedly reduced [Ca^2 +^]_o_-induced vasorelaxations. Since charybdotoxin (CbTX) blocks BKCa and IKCa channels we investigated the role of IKCa channel activity by pre-treating vessel segments with 100 nM IbTX and 100 nM CbTX. Fig. 5A shows that co-application of these two agents induced an additional inhibition of [Ca^2 +^]_o_-mediated relaxations compared to IbTX alone. In fact, in the presence of IbTx and CbTx increasing [Ca^2 +^]_o_ between 1 mM and 5 mM induced an increase in pre-contracted tone (Fig. 5A). To confirm that the additive inhibitory effect of CbTX and IbTX co-application was not due to enhanced BKCa blockade, a control experiment was performed in which vessels were pre-treated with 200 nM IbTX. Importantly, this did not have any additional inhibitory effect on [Ca^2 +^]_o_-induced vasorelaxations compared with vessels pre-treated with 100 nM IbTX (Fig. 5A).

Pre-treatment with 10 μM linopirdine, a selective Kv7 blocker, only reduced vasorelaxations produced by 6 mM [Ca^2 +^]_o_, whilst 100 nM apamin and 10 μM glibenclamide, blockers of SKCa and K_ATP_ channels respectively, and 10 μM indomethacin, a cycloxygenase inhibitor, had no effect on [Ca^2 +^]_o_-induced relaxations of pre-contracted tone (Fig. 5B).

These findings suggest that BKCa- and IKCa-mediated hyperpolarisations of VSMCs may make essential contributions to CaSR-evoked vasorelaxations. We tested this proposal by studying the effect of [Ca^2 +^]_o_ on pre-contracted tone induced by 60 mM KCl in vessel segments containing a functional endothelium. In these conditions, the membrane potential of VSMCs is clamped at a fixed membrane potential of about − 20 mV, evoking vasoconstrictions that are entirely mediated by voltage-dependent calcium-channels (Supplementary Figure S5). Fig. 5C shows that increasing [Ca^2 +^]_o_ from 1 mM to 6 mM failed to induce relaxations in KCl pre-contracted vessels, and instead, increasing [Ca^2 +^]_o_ potentiated contractility.

In a final series of functional experiments, we investigated the combined effects of NO, and BKCa and IKCa channels on [Ca^2 +^]_o_-induced relaxations by using L-NAME to inhibit eNOS and CbTX to block BKCa and IKCa channels. As expected, Fig. 5D illustrates that co-application of 300 μM L-NAME and 100 nM CbTX abolished [Ca^2 +^]_o_-induced relaxations of pre-contracted tone. In fact, in the presence of L-NAME and CbTX responses to [Ca^2 +^]_o_ were similar to those induced in the absence of a functional endothelium (Fig. 1A and B), with [Ca^2 +^]_o_ inducing a marked potentiation of pre-contracted tone.

These results suggest that activation of BKCa and IKCa channel induces hyperpolarisations which have significant roles in mediating CaSR-induced relaxations in rabbit mesenteric arteries. Since BKCa and IKCa channels are selectively expressed in VSMCs and ECs respectively, it is likely that CaSRs induce BKCa channel activity through the NO/PKG-mediated pathway described above whereas CaSRs activate IKCa channels via an unknown mechanism.

### 3.4 Stimulation of CaSRs activates IKCa currents

To provide further evidence that stimulation of CaSRs induces activation of IKCa channels in ECs, we carried out electrophysiological recordings using whole-cell and perforated patch configurations of the patch clamp technique from freshly isolated rabbit mesenteric artery ECs.

**Fig. 6:**
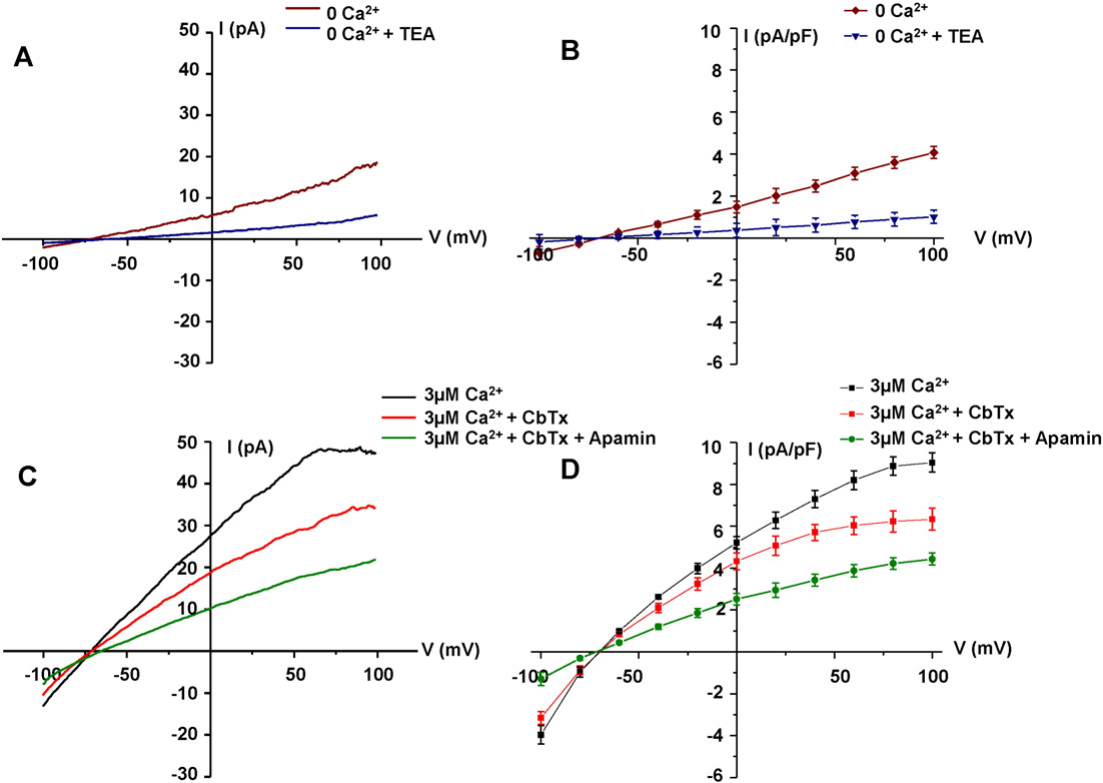
IKCa and SKCa currents in freshly isolated ECs. (A) Representative trace and (B) mean I/V relationship of whole-cell patch recordings showing a TEA-sensitive current present in the absence of [Ca^2 +^]_i_, (C) and (D) show that inclusion of 3 µM free Ca^2 +^ in the patch pipette solution evoked currents which were inhibited by CbTX (100 nM) and apamin (300 nM). All points are from n = 6 patches from at least n = 3 animals.

Fig. 6A and B show that in the absence of Ca^2 +^ in the patch pipette solution, application of voltage ramps from − 100 mV to + 100 mV from a holding potential of − 60 mV evoked a linear mean whole-cell current which reversed near to the equilibrium potential for K^+^ ions (E_K_ is − 80 mV), and was inhibited by the non-selective voltage-gated K^+^ channel blocker tetraethylammonium (TEA). Fig.6C and D shows that inclusion of 3 μM free Ca^2 +^ in the patch pipette solution evoked a much larger mean whole-cell current, which displayed inward rectification at positive potentials, and reversed close to E_K_. Mean whole-cell currents induced by 3 μM free Ca^2 +^ were reduced by 100 nM CbTX and further inhibited by co-applications of 100 nM CbTX with 100 nM apamin. In these latter conditions, the mean whole-cell current resembled the linear current induced in the absence of Ca^2 +^. These findings show that rabbit mesenteric ECs express functional native IKCa and SKCa channels as previously described in other native ECs [24], [33], [34], [35], [36], [37].

**Fig. 7:**
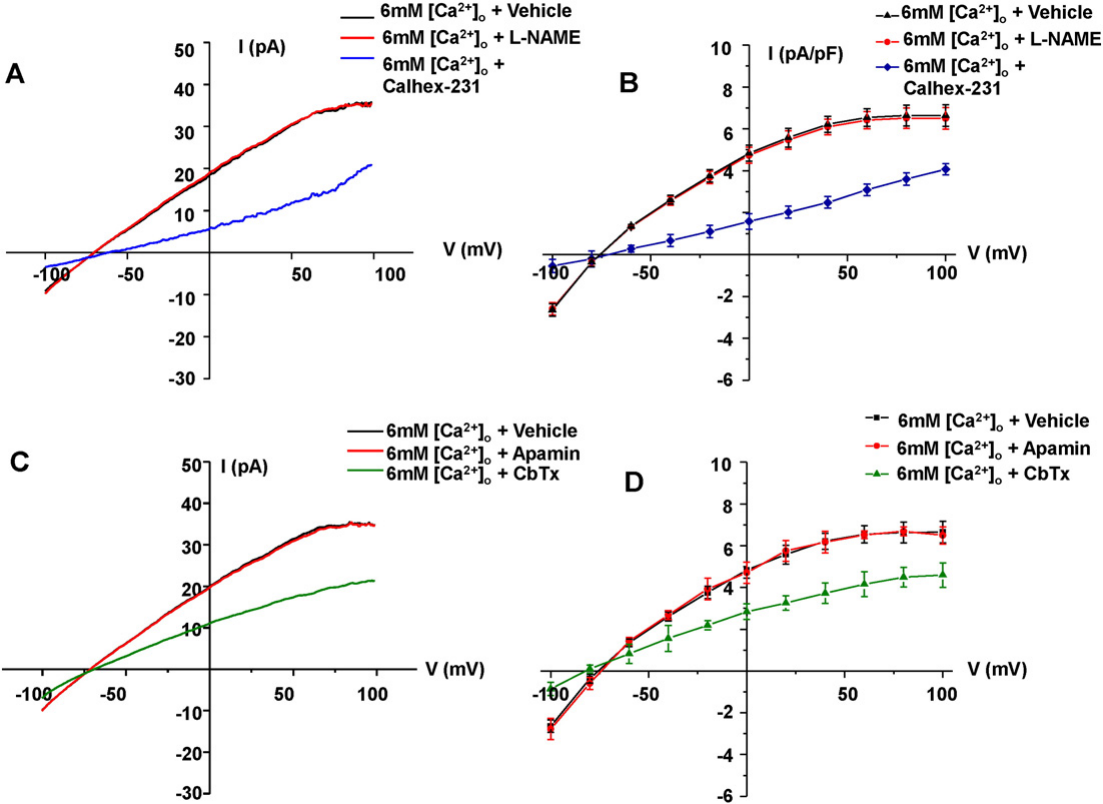
[Ca^2 +^]_o_-induced IKCa currents in freshly isolated ECs. (A) Representative trace and (B) the mean I/V relationships of perforated-patch recordings showing that increasing [Ca^2 +^]_o_ from 1 mM to 6 mM evoked currents which were inhibited by Calhex-231 (3 µM) but were unaffected by L-NAME (300 µM). (C) and (D) show that [Ca^2 +^]_o_-induced currents were also inhibited by the addition of CbTX (100 nM) but were unaffected by apamin (300 nM). All points are from n = 6 patches from at least n = 3 animals.

Fig. 7A and B demonstrate that increasing [Ca^2 +^]_o_ from 1 mM to 6 mM evoked a mean macroscopic current using the perforated patch configuration which had similar inward rectification at positive potentials and reversal potential to the mean whole-cell current induced by 3 μM free Ca^2 +^. Fig. 7A and B shows that the mean [Ca^2 +^]_o_-induced current was inhibited by application of 3 μM Calhex-231 but was not affected by 300 μM L-NAME. Moreover, Fig. 7C and D shows that the mean [Ca^2 +^]_o_-induced current was reduced by 100 nM CbTX but was unaffected by 100 nM apamin. These results indicate that stimulation of CaSRs evokes native IKCa currents in rabbit mesenteric artery ECs via a NO-independent pathway.

## 4 Discussion

This study shows that stimulation of CaSRs by increasing [Ca^2 +^]_o_ induces endothelium-dependent relaxations of pre-contracted rabbit mesenteric arteries. We propose that these CaSR-induced vasorelaxations are mediated by two separate pathways initiated in ECs: NO generation leading to stimulation of a PKG-mediated pathway and opening of BKCa channels in VSMCs, and stimulation of IKCa channel activity which is likely to induce endothelium-derived hyperpolarisations.

### 4.1 CaSR-mediated relaxations are endothelium-dependent

Increasing [Ca^2 +^]_o_ between 1 mM and 6 mM induced a concentration-dependent relaxation of pre-contracted tone in rabbit mesenteric arteries, which was markedly inhibited by the calcilytic Calhex-231 and abolished by removal of a functional endothelium. In contrast, increasing [Ca^2 +^]_o_ potentiated pre-contracted tone in endothelium-removed vessel segments. As predicted from these findings, CaSRs were expressed at the plasma membrane of freshly isolated mesenteric artery ECs and VSMCs. These results suggest that in the endothelium-intact artery, CaSR-mediated vasorelaxations are the dominant physiological phenomenon.

These results are also consistent with earlier studies showing that CaSR mRNA and protein are expressed in ECs [10], [12], [14], [16], [38], [39] and VSMCs [12], [18], [19], [20], [21], [22], [24], [40], [41], [42], and that stimulation of these CaSRs induces endothelium-dependent relaxations [12], [13], [14], [16] and endothelium-independent vasoconstrictions [17], [18], respectively. Increasing [Ca^2 +^]_o_ has also been shown to induce CaSR-mediated biphasic effects on pre-contracted tone, with an initial contraction followed by relaxation [9], [43]. CaSRs are also reported to be expressed on perivascular nerves, and stimulation of these receptors is proposed to induce the release of a nerve derived hyperpolarising factor (NDHF) which activates BKCa channels in VSMCs to induce hyperpolarisation and vasorelaxation [6], [7], [44], [45], [46]. The present study indicates a significant role for the endothelium in mediating CaSR-induced vasorelaxations, which suggests that perivascular nerves are unlikely to be involved. It is not known why there are differences between the present work and these earlier findings, although recent evidence indicates that capsaicin used to provide evidence for the involvement of perivascular nerves may also induce vasorelaxations through activation of TRPV1 channels expressed in ECs [47], [48], and the identity of the proposed NDHF is currently unknown. Moreover, there may be species differences in CaSR-mediated vascular effects.

### 4.2 NO production contributes to CaSR-induced endothelium-dependent relaxations

[Ca^2 +^]_o_-induced vasorelaxations of pre-contracted tone were reduced by inhibitors of eNOS, GC, PKG, and BKCa channels. These data provide strong evidence that stimulation of CaSRs induces the well-characterised NO–GC–PKG signalling cascade system, which couples to opening of BKCa channels in VSMCs leading to hyperpolarisation and vasorelaxation. A direct stimulatory action of NO on BKCa channels may also contribute to CaSR-mediated vasorelaxations [49], [50]. Further evidence that NO production plays a pivotal role in CaSR-mediated vasorelaxations was provided by experiments showing that increasing [Ca^2 +^]_o_ induced NO production in isolated ECs loaded with fluorescent NO indicator DAF-FM, which was inhibited by Calhex-231 and L-NAME. An interesting observation was that basal DAF-FM fluorescence recorded in the presence of 1 mM [Ca^2 +^]_o_ was also inhibited by Calhex-231 and L-NAME. This provides evidence that physiological plasma [Ca^2 +^]_o_ levels may mediate resting vascular tone through stimulation of CaSRs and induce basal production of NO.

Our data are consistent with earlier data indicating a potential role for NO in CaSR-mediated responses in ECs and in other cell types [10], [11], [12], [13], [51], [52]. In cultured human aortic endothelial cells, Ziegelstein et al. [10] demonstrated a CaSR-mediated rise in intracellular [Ca^2 +^] and subsequent NO generation, but only when CaSRs were stimulated with spermine and not with other CaSR agonists such as Ca^2 +^, Gd^3 +^, or neomycin. In contrast, this study was able to demonstrate Ca^2 +^-induced CaSR activation and resulting NO production. Potentially, this difference may reflect variability in CaSR-signalling between cultured ECs and freshly isolated native ECs. In a later study using the calcimimetic AMG 073, Smajilovic et al. [11] observed dose-dependent vasorelaxations of pre-contracted rat aorta, with a significant L-NAME-sensitive component present. Importantly, however, the authors did not rule out whether this response was CaSR-mediated, and other studies have indeed demonstrated that several calcimimetic compounds have CaSR-independent effects that may explain their vascular actions. For example, the calcimimetic R-568 was shown to directly induce NO production in cultured human umbilical vein ECs without involving CaSR activation, and the calcimimetic NPS R-467 directly activates non-specific cation channels [61]. The clinically-used mimetic cinacalcet and calindol have also been shown to directly block voltage-gated calcium channels and activate VSMC K^+^ channels [9]. Accordingly, our data using the endogenous CaSR ligand in freshly isolated native ECs offer important insights into the functional role of vascular CaSRs in vascular tone regulation.

### 4.3 CaSR-induced activation of IKCa channels in ECs contributes to relaxations

Co-application of 100 nM IbTX together with 100 nM CbTX produced a markedly greater inhibition of [Ca^2 +^]_o_-induced vasorelaxations compared to 100 nM IbTX alone. IbTX is a selective BKCa blocker whereas CbTX blocks both BKCa and IKCa, and therefore this additive effect is most likely due to CaSR-mediated activation of IKCa channels which are expressed in ECs but not VSMCs. In addition, increasing [Ca^2 +^]_o_ from 1 mM to 6 mM evoked macroscopic K^+^ conductances, which had similar properties to previously described IKCa channels in vascular ECs [23] and were inhibited by Calhex-231 and CbTX. Previous studies have used TRAM-34 to block IKCa channels and thereby demonstrate IKCa involvement in endothelial responses [14], [15], [53], [54]. However, this study did not use TRAM-34 since it also inhibits non-selective cation channels [55], which may be important for generating the observed CaSR responses. The present work and others [24], [33] also show that SKCa channel activity is present in ECs, although it seems unlikely that SKCa channels have a role in CaSR-mediated vasorelaxations as the selective SKCa blocker apamin had no effect on CaSR-induced vasorelaxations or K^+^ channel activity. The selective activation of IKCa over SKCa following CaSR activation potentially reflects the functional co-localisation of IKCa and CaSRs in the EC plasma membrane [14], although the precise mechanism responsible for CaSR-induced IKCa activation remains unclear and is the subject of ongoing investigation.

Co-application of L-NAME and CbTX produced an additional inhibitory effect on CaSR-induced relaxations compared to applications of L-NAME alone. Moreover, L-NAME did not affect CaSR-induced IKCa channel activity in isolated ECs. These findings indicate that CaSR-mediated vasorelaxations are unlikely to involve interactions between NO-mediated pathways and IKCa channels, which are consistent with earlier findings showing that NO is involved in activating SKCa but not IKCa channels in ECs [56]. It was apparent that following inhibition of NO- and IKCa-mediated pathways, increasing [Ca^2 +^]_o_ potentiated pre-contracted tone, with a similar magnitude to that observed with removal of a functional endothelium. This further indicates that CaSR-induced endothelium-dependent vasorelaxations are mainly produced through NO- and IKCa channel-mediated pathways. In future studies it will be interesting to investigate if the contribution of these two pathways to CaSR-mediated vasorelaxations differs at different [Ca^2 +^]_o_, and with different vascular beds and order branches.

Our results also indicate a role for Kv7 channels in CaSR-mediated relaxations at higher [Ca^2 +^]_o_, as the pan Kv7 blocker linopirdine produce a small by significant reduction in these responses. Heteromeric Kv7.4/7.5 channels and other unidentified Kv channels expressed in VSMCs are reported to mediate relaxations induced by NO [27], [57], [58], [59], [60]. It is therefore possible that activation of Kv7.4/7.5 channels via a CaSR-mediated NO-mediated pathway may contribute to the observed vasorelaxations.

### 4.4 Possible significance of findings

The present work clearly indicates that endothelium-dependent vasorelaxations mediated by CaSRs contribute to the regulation of vascular tone. Indeed even at plasma Ca^2 +^ levels of 1–2 mM, CaSR-induced vasorelaxations occur, suggesting that endothelial CaSRs may sense free Ca^2 +^ ions in the blood as they pass over the luminal EC surface. Furthermore, active Ca^2 +^ extrusion from adjacent contracting vascular myocytes, may cause the localised [Ca^2 +^]_o_ to be much higher within the tight space between the two cell layers than it is within the circulating blood [1], [3]. This paracrine Ca^2 +^ signalling may then help to amplify the contribution of vascular CaSRs to vascular tone regulation [62].

## 5 Conclusions

Stimulation of CaSRs induces endothelium-dependent vasorelaxations which are mediated by two separate pathways involving production of NO and activation of IKCa channels. NO stimulates PKG leading to BKCa activation in vascular smooth muscle cells, whereas IKCa activity contributes to endothelium-derived hyperpolarisations.

## Funding

This work was supported by a British Heart Foundation PhD Studentship for H. Z. E. Greenberg (FS/13/10/30021 to A.P.A); and by the Biotechnology and Biological Sciences Research Council (BB/J007226/1 to A.P.A).

## Conflict of interest

None declared.

## Figures and Tables

**Table 1:** The effect of the various inhibitors tested on [Ca^2 +^]_o_-induced vasorelaxations.

	EC_50_ (mM)	E_max_ (%)	n
Controls	2.2 ± 0.04	96 ± 3.4	8
-Endothelium	−	−	8
+ Calhex-231 (μM)	4 ± 0.51⁎⁎⁎⁎	54.6 ± 4.6⁎⁎	4
+ L-NAME (300 μM)	4.1 ± 0.01⁎⁎⁎⁎	8.3 ± 11⁎⁎⁎⁎	7
+ ODQ (10 μM)	3.1. ± 0.59⁎⁎⁎	30.8 ± 14⁎⁎⁎	4
+ KT5283 (1 μM)	4.5 ± 0.42⁎⁎⁎	53.4 ± 12⁎⁎	4
+ IbTx (100 nM)	4.3 ± 0.21⁎⁎⁎⁎	51.9 ± 5.1⁎⁎⁎	4
+ IbTx (100 nM) + CbTx (100 nM)	5.1 ± 0.03⁎⁎⁎⁎	15 ± 11.7⁎⁎⁎⁎	5
+ IbTX (200 nM)	4.1 ± 0.34⁎⁎⁎⁎	45 ± 7.2⁎⁎⁎	5
+ Apamin (100 nM)	2.1 ± 0.01	93 ± 3.3	4
+ Linopirdine (10 μM)	2.1 ± 0.22	72 ± 3.3⁎⁎	4
+ Indomethacin(10 μM)	2.3 ± 0.05	92 ± 3.5	5
+ Glibenclamide	2.1 ± 0.31	91 ± 4.6	4
+ L-NAME (300 μM) + CbTx(100 nM)	−	−	5

EC_50_ and E_max_ values were compared with Student's t-test vs. corresponding controls. n = animals used, with at least 3 vessel segments per animal.

⁎⁎ p < 0.01.

⁎⁎⁎ p < 0.001.

⁎⁎⁎⁎ p < 0.0001.

## Appendix A Supplementary data

**Figure S1.** Effect of [Ca^2 +^]_o_ on U46619 pre-contracted tone of rabbit mesenteric arteries. (*A*) Representative traces showing the effect of [Ca^2 +^]_o_ on pre-contracted tone induced by 300nM U46619 on endothelium-intact (Left trace) and endothelium-removed vessels (Right trace). (*B*) Mean data showing the effect of [Ca^2 +^]_o_ on U46619-induced pre-contracted vessels. Each point is from n = 4 animals, with at least n = 3 vessel segments per animal. **p < 0.01, ***p < 0.001. **Figure S2** Effect of Calhex-231 on CCh-mediated relaxation of pre-contracted tone. Pre-treatment with 3 μM Calhex-231 had no effect on CCh-induced concentration-dependent relaxations of pre-contracted tone. All points are from n = 4 animals, with at least n = 3 segments per animal. **Figure S3** Effect of KT5823 on pre-contracted tone. (A) Representative images and (B) mean data showing that 1 μM KT5823 induced about a 25% relaxation of pre-contracted tone which was inhibited by removal of a functional endothelium and co-application of 100 nM CbTx and 100 nM apamin but not by application of 300 μM L-NAME. All points are from n = 3 animals, with at least n = 3 segments per animal. * p < 0.05. **Figure S4.** Effect of CCh on NO production in isolated ECs measured using DAF-FM fluorescence. (A) Mean data showing that CCh increased basal fluorescence which was inhibited pre-treating cells with 300 μM L-NAME but not by (B) 3 μM Calhex-231. n = at least 50 cells from n = 4 animals. **p < 0.01, ***p < 0.001. **Figure S5.** Effect of NiCardipine on KCl-precontracted tone. (A) Representative trace and (B) Bar graph of mean data showing that 1 μM NiCardipine (voltage-gated calcium channel inhibitor) completely abolishes 60 mM KCl-induced pre-contracted tone. Bars and error bars are mean values ± s.e.m. from n = 4 animals, with at least n = 3 vessel segments from each animal. ****p < 0.001.

## References

[1] Brown E.M., MacLeod R.J. (2001). Extracellular calcium sensing and extracellular calcium signalling. Physiol. Rev..

[2] Smajilovic S., Yano S., Jabbari R., Tfelt-Hansen J. (2011). The calcium-sensing receptor and calcimimetics in blood pressure modulation. Br. J. Pharmacol..

[3] Hofer A.M. (2005). Another dimension to calcium signalling: a look at extracellular calcium. J. Cell Sci..

[4] Weston A.H., Geraghty A., Egner I., Edwards G. (2011). The vascular extracellular calcium-sensing receptor: an update. Acta Physiol..

[5] Reid I.R. (2013). Cardiovascular effects of calcium supplements. Nutrients.

[6] Bukoski R.D., Bian K., Wang Y., Mupanomunda M. (1997). Perivascular sensory nerve Ca^2 +^ receptor and Ca^2 +^-induced relaxation of isolated arteries. Hypertension.

[7] Ishioka N., Bukoski R.D. (1999). A role for N-arachidonylethanolamine (anandamide) as the mediator of sensory nerve-dependent Ca^2 +^-induced relaxation. J. Pharmacol. Exp. Ther..

[8] Awumey E.M., Hill S.K., Diz D.I., Bukoski R.D. (2008). Cytochrome P-450 metabolites of 2-arachidonoylglycerol play a role in Ca^2 +^-induced relaxation of rat arteries. Am. J. Physiol. Heart Circ. Physiol..

[9] Thakore P., Ho W.-S.V. (2011). Vascular actions of calcimimetics: role of Ca^2 +^-sensing receptors versus Ca^2 +^ influx through L-type Ca^2 +^ channels. Br. J. Pharmacol..

[10] Ziegelstein R.C., Xiong Y., He C., Hu Q. (2006). Expression of a functional extracellular calcium-sensing receptor in human aortic endothelial cells. Biochem. Biophys. Res. Commun..

[11] Smajilovic S., Sheykhzade M., Holmegard H.N., Hanuso S., Tfelt-Hansen J. (2007). Calcimimetric, AMG, induces relaxation on isolated rat aorta. Vasc. Pharmacol..

[12] Loot A.E., Pierson I., Syzonenko T., Elgheznawy A., Randriamboavonjy V., Zivkovic A. (2013). Ca^2 +^-sensing receptor cleavage by calpain partially accounts for altered vascular reactivity in mice fed a high-fat diet. J. Cardiovasc. Pharmacol..

[13] Awumey E.M., Bridges L.E., Williams C.L., Diz D.I. (2013). Nitric-oxide synthase knockout modulates Ca^2 +^-sensing receptor expression and signalling in mouse mesenteric arteries. J. Pharmacol. Exp. Ther..

[14] Weston A.H., Absi M., Ward D.T., Ohanian J., Dodd R.H., Dauban P. (2005). Evidence in favour of a calcium-sensing receptor in arterial endothelial cells: studies with calindol and calhex-231. Circ. Res..

[15] Dora K.A., Gallagher N.T., McLeish A., Garland C.J. (2008). Modulation of endothelial cell K_Ca_3.1 channels during endothelium-derived hyperpolarisation factor signalling in mesenteric resistance arteries. Circ. Res..

[16] Weston A.H., Absi M., Harno E., Geraghty A.R., Ward D.T., Ruat M. (2008). The expression and functional of Ca^2 +^-sensing receptors in rat mesenteric artery; comparative studies using a model of type II diabetes. Br. J. Pharmacol..

[17] Wonneberger K., Scofield M.A., Wangemann P. (2000). Evidence for a calcium-sensing receptor in the vascular smooth muscle cells of the spiral modiolar artery. J. Membr. Biol..

[18] Li G.-W., Wang Q.-S., Hao J.-H., Xing W.-J., Guo J., Li H.-Z. (2011). The functional expression of extracellular calcium-sensing receptor in rat pulmonary artery smooth muscle cells. J. Biomed. Sci..

[19] Smajilovic S., Hansen J.L., Christoffersen T.H.E., Lewin E., Sheokh S.P., Terwilliger E.F. (2006). Extracellular calcium sensing in rat aortic vascular smooth muscle cells. Biochem. Biophys. Res. Commun..

[20] Molostvov G., James S., Fletcher S., Bennett J., Lehnert H., Bland R. (2007). Extracellular calcium-sensing receptor is functionally expressed in human artery. Am. J. Physiol. Renal Physiol..

[21] Molostvov G., Fletcher S., Bland R., Zehnder D. (2008). Extracellular calcium-sensing receptor mediated signalling is involved in human vascular smooth muscle cell proliferation and apoptosis. Cell. Physiol. Biochem..

[22] Li G.-W., Xing W.-J., Bai S.-Z., Hao J.-H., Guo J., Li H.-Z. (2010). The calcium-sensing receptor mediates hypoxia-induced proliferation of rat pulmonary artery smooth muscle cells through MEK1/ERK1,2 and PI3K pathways. Basic Clin. Pharmacol. Toxicol..

[23] Mulvany M.J., Halpern W. (1977). Contractile properties of small arterial resistance vessels in spontaneously hypertensive and normotensive rats. Circ. Res..

[24] Ledoux J., Bonev A.D., Nelson M.T. (2008). Ca^2 +^-activated K^+^ channels in murine endothelial cells: block by intracellular calcium and magnesium. J. Gen. Physiol..

[25] Petrel C., Kessler A., Maslah F., Dauban P., Dodd R.H., Rognan D. (2003). Modelling and mutagenesis of the binding site of Calhex 231, a novel negative allosteric modulator of the extracellular Ca^2 +^-sensing receptor. J. Biol. Chem..

[26] Francis S.H., Busch J.L., Corbin J.D., Sibley D. (2010). cGMP-dependent protein kinases and cGMP phosphodiestereases in nitric oxide and cGMP action. Pharmacol. Rev..

[27] Dora K.A., Garland C.J., Kwan H.Y., Yao X. (2001). Endothelial cell protein kinase G inhibits release of EDHF through a PKG-sensitive cation channel. Am. J. Physiol. Circ. Physiol..

[28] Pittner J., Liu R., Brown R., Wolgast M., Persson A.E. (2003). Visualization of nitric oxide production and intracellular calcium in juxtamedullary afferent arteriolar endothelial cells. Acta Physiol. Scand..

[29] Edwards G., Feletou M., Weston A.H. (2010). Endothelium-derived hyperpolarising factor and associated pathways: synopsis. Pflugers Arch..

[30] Garland C.J., Hiley C.R., Dora K.A. (2011). EDHF: spreading the influence of the endothelium. Br. J. Pharmacol..

[31] Brayden J.E. (2002). Functional roles of KATP channels in vascular smooth muscle. Clin. Exp. Pharmacol. Physiol..

[32] Jepps T.A., Olesen S.P., Greenwood I.A. (2013). One man's side effect is another man's therapeutic opportunity: targeting Kv7 channels in smooth muscle disorders. Br. J. Pharmacol..

[33] Burnham M.P., Bychkov R., Feletou M., Richards G.R., Vanhoutte P.M., Weston A.H. (2002). 2002. Characterization of an apamin-sensitive small-conductance Ca^2 +^-activated K^+^ channel in porcine coronary artery endothelium: relevance to EDHF. Br. J. Pharmacol..

[34] Bychkov R., Burnham M.P., Richards G.R., Edwards G., Weston A.H., Feletou M. (2002). Characterization of a charybdotoxin-sensitive intermediate conductance Ca^2 +^-activated K^+^ channel in porcine coronary endothelium: relevance to EDHF. Br. J. Pharmacol..

[35] Eichler I., Wibawa J., Grgic I., Knorr A., Brakemeier S., Pries A.R. (2003). Selective blockade of endothelial Ca^2 +^-activated small- and intermediate-conductance K^+^-channels suppresses EDHF-mediated vasodilation. Br. J. Pharmacol..

[36] Si H., Heyken W.T., Wolfle S.E., Tysiac M., Schubert R., Grgic I. (2006). Impaired endothelium-derived hyperpolarizing factor-mediated dilations and increased blood pressure in mice deficient of the intermediate-conductance Ca^2 +^-activated K^+^ channel. Circ. Res..

[37] Taylor M.S., Bonev A.D., Gross T.P., Eckman D.M., Brayden J.E., Bond C.T. (2003). Altered expression of small-conductance Ca^2 +^-activated K^+^ (SK3) channels modulates arterial tone and blood pressure. Circ. Res..

[38] Berra Romani R., Raqeeb A., Laforenza U., Scaffino M.F., Moccia F., Avelino-Cruz J.E. (2009). Cardiac microvascular endothelial cells express a functional Ca^2 +^-sensing receptor. J. Vasc. Res..

[39] Bonomini M., Giardinelli A., Morabito C., Di Silvestre S., Di Cesare M., Di Pietro N. (2012). Calcimimetic R-568 and its enantiomer S-568 increase nitric oxide release in human endothelial cells. PLoS ONE.

[40] Alam M.U., Kirton J.P., Wilkinson F.L., Towers E., Sinha S., Rouhi M. (2009). Calcification is associated with loss of functional calcium-sensing receptor in vascular smooth muscle cells. Cardiovasc. Res..

[41] Chow J.Y., Estrema C., Orneles T., Dong X., Barrett K.E., Dong H. (2011). Calcium-sensing receptor modulates extracellular Ca^2 +^ entry via TRPC-encoded receptor-operated channels in human aortic smooth muscle cells. Am. J. Physiol. Cell Physiol..

[42] Yamamura A., Guo Q., Yamamura H., Zimnicka A.M., Pohl N.M., Smith K.A. (2012). Ca^2 +^-sensing receptor function in idiopathic pulmonary arterial hypertension. Circ. Res..

[43] Ohanian J., Gatfield K.M., Ward D.T., Ohanian V. (2005). Evidence for a functional calcium-sensing receptor that modulates myogenic tone in rat subcutaneous small arteries. Am. J. Physiol. Heart Circ. Physiol..

[44] Mupanomunda M., Wang Y., Bukoski R.D. (1998). Effects of chronic sensory denervation on Ca^2 +^-induced relaxation of isolated mesenteric resistance arteries. Am. J. Physiol..

[45] Wang Y., Bukoski R.D. (1998). Distribution of the perivascular nerve Ca^2 +^ receptor in rat arteries. Br. J. Pharmacol..

[46] Bukoski R.D., Bátkai S., Járai Z., Wang Y., Offertaler L., Jackson W.F. (2002). CB_1_ receptor antagonist SR141716A inhibits Ca^2 +^-induced relaxation in CB_1_ receptor-deficient mice. Hypertension.

[47] Poblete I., Orliac M., Briones R., Adler-Graschinsky E., Huidobro-Toro J. (2005). Anandamide elicits an acute release of nitric oxide through endothelial TRPV1 receptor activation in the rat arterial mesenteric bed. J. Physiol..

[48] Yang D., Luo Z., Ma S., Wong W., Ma L., Zhong J. (2010). Activation of TRPV1 by dietary capsaicin improves endothelium-dependent vasorelaxation and prevents hypertension. Cell Metab..

[49] Bolotina V.M., Najibi S., Palacino J.J., Pagano P.J., Cohen R.A. (1994). Nitric oxide directly activates calcium-dependent potassium channels in vascular smooth muscle. Nature.

[50] Mistry D.K., Garland C.J. (1998). Nitric oxide (NO)-induced activation of large conductance Ca^2 +^-dependent K^+^ channels (BK(Ca)) in smooth muscle cells isolated from the rat mesenteric artery. Br. J. Pharmacol..

[51] Dal Pra I., Chiarini A., Nemeth E., Armato U., Whitfield J. (2005). Roles of Ca^2 +^ and the Ca^2 +^-sensing receptor (CASR) in the expression of inducible NOS (nitric oxide synthase)-2 and its BH4 (tetrahydrobiopterin)-dependent activation in cytokine-stimulated adult human astrocytes. J. Cell. Biochem..

[52] Tfelt-Hansen J., Ferreira A., Yano S., Kanuparthi D., Romero J.R., Brown E.M. (2005). Calcium-sensing receptor activation induces nitric oxide production in H-500 Leydig cancer cells. Am. J. Physiol. Endocrinol. Metab..

[53] Wulff H., Miller M.J., Haensel W., Grissner S., Cahalan M.D., Chandy K.G. (2000). Design of potent and selective inhibitor of the intermediate-conductance Ca^2 +^-activated K^+^ channel, IKCa1: a potential immunosuppressant. Proc. Natl. Acad. Sci. U. S. A..

[54] Wulff H., Kolski-Andreaco A., Sankaranarayanan A., Sabatier J.M., Shakkottai V. (2007). Modulators of small- and intermediate-conductance calcium-activated potassium channels and their therapeutic indications. Curr. Med. Chem..

[55] Schilling T., Eder C. (2007). TRAM-34 inhibits nonselective cation channels. Pflugers Arch..

[56] McNeish A., Sandow S., Neylon C., Chen M., Dora K.A., Garland C.J. (2006). Evidence for involvement of both IKCa and SKCa channels in hyperpolarizing responses of the rat middle CaSRebral artery. Stroke.

[57] Lovren F., Triggle C. (2000). Nitric oxide and sodium nitroprusside-induced relaxation of the human umbilical artery. Br. J. Pharmacol..

[58] McNeish A., Altayo F., Garland C.J. (2010). Evidence both L-type and non-L-type voltage-dependent calcium channels contribute to cerebral artery vasospasm following loss of NO in the rat. Vasc. Pharmacol..

[59] Ooi L., Gigout S., Pettinger L., Gamper N. (2013). Triple cysteine module within M-type K + channels mediates reciprocal channel modulation by nitric oxide and reactive oxygen species. J. Neurosci..

[60] Stott J.B., Barrese V., Jepps T.A., Leighton E.V., Greenwood I.A. (2015). Contribution of Kv7 channels to natriuretic peptide mediated vasodilation in normal and hypertensive rats. Hypertension.

[61] Straub S.G., Kornreich B., Oswald R.E., Nemeth E.F., Sharp G.W. (2000). The calcimimetic R-467 potentiates insulin secretion in pancreatic beta cells by activation of a nonspecific cation channel. J. Biol. Chem..

[62] Schepelmann M., Yarova P., Lopez-Fernandez I., Davies T., Brennan S., Edwards P., Aggarwal A., Graca J., Rietdorf K., Matchkov V., Fenton R., Chang W., Krssak M., Stewart A., Broadley K., Ward D., Price S., Edwards D., Kemp P., Riccardi D. (2015). The vascular Ca^2 +^-sensing receptor regulates blood vessel tone and blood pressure. Am. J. Physiol. Cell Physiol..

