# Supplementary material for: Stimulation of calcium-sensing receptors induces endothelium-dependent vasorelaxations via nitric oxide production and activation of IKCa channels
Source: Vascul Pharmacol. 2016 May;80:75–84. doi: 10.1016/j.vph.2016.01.001 (PMC4830458; doi:10.1016/j.vph.2016.01.001)

Supplementary Figure 1

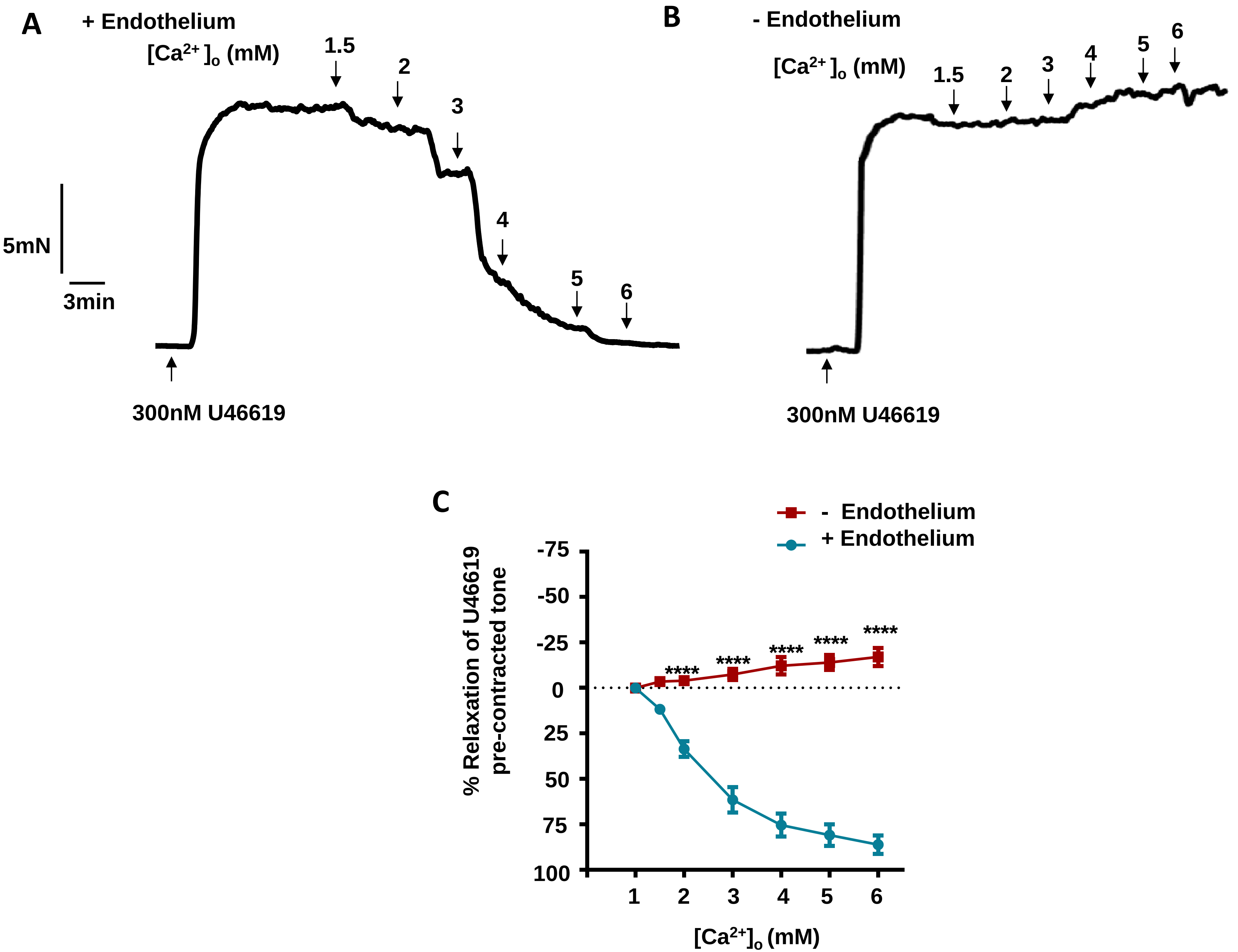

Supplementary Figure 2

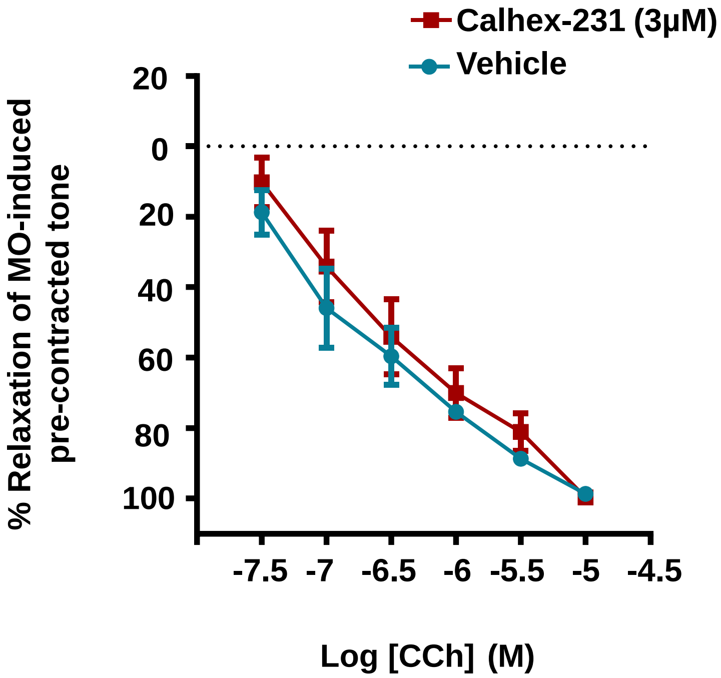

Supplementary Figure 3

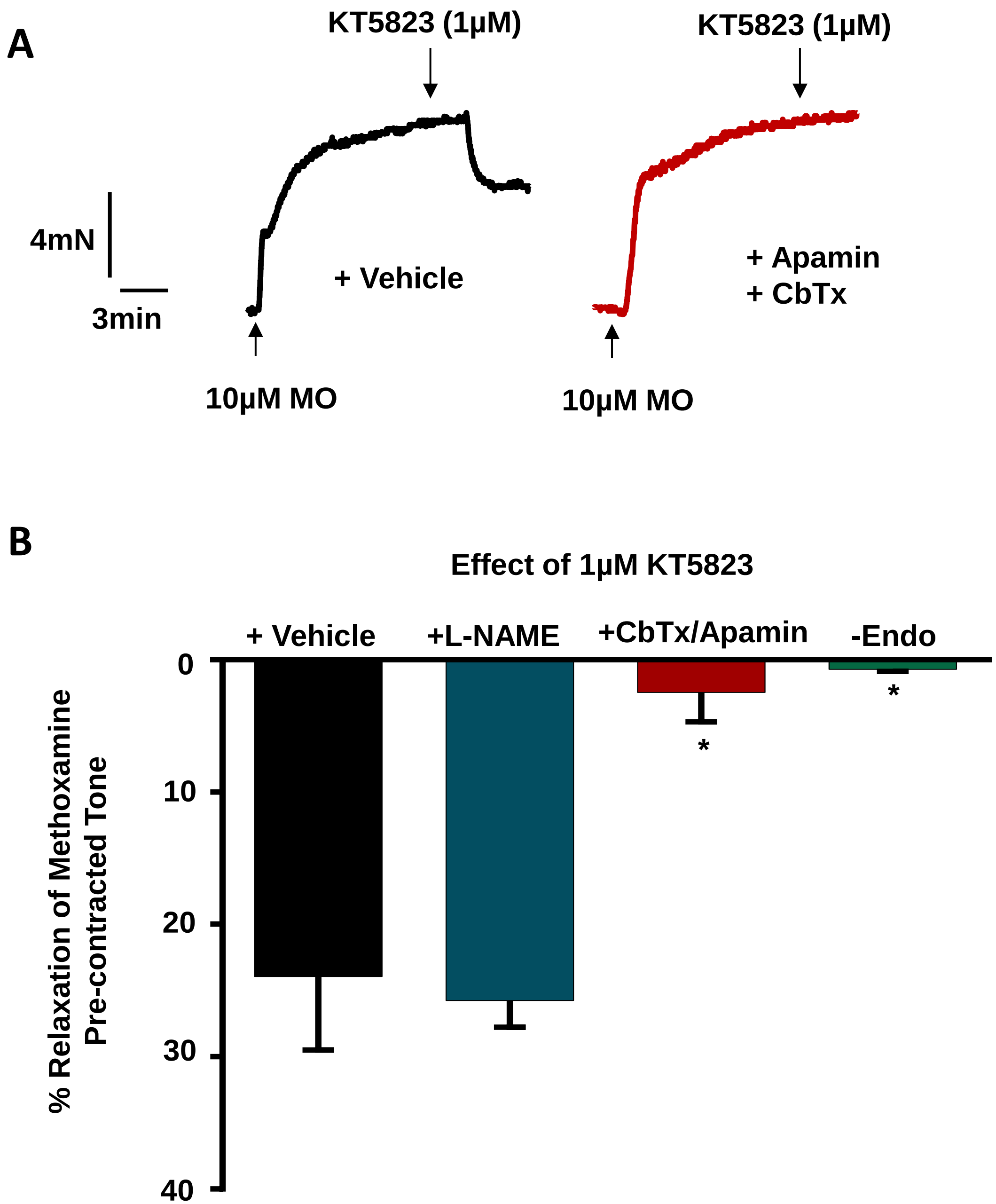

Supplementary Figure 4

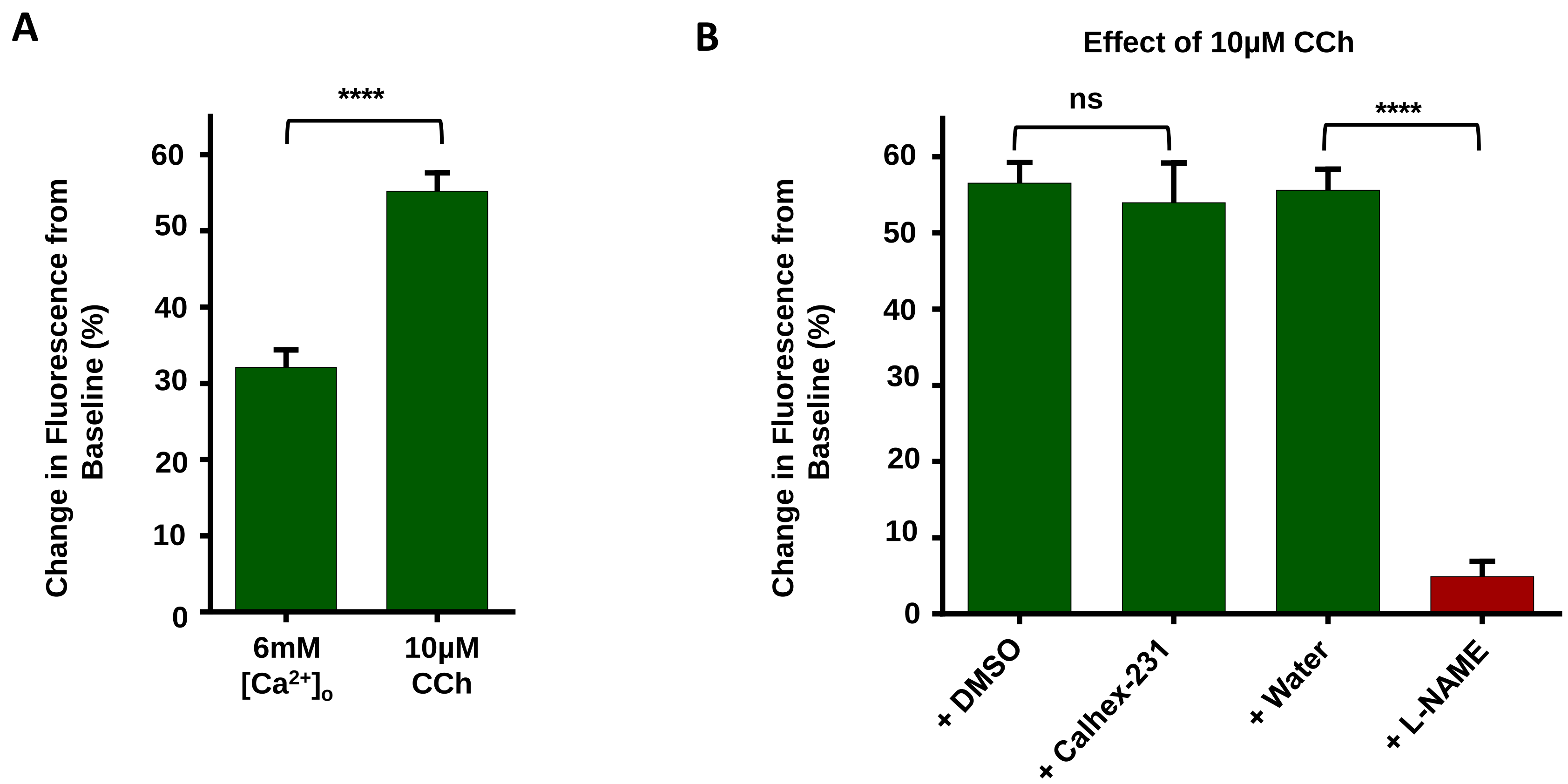

Supplementary Figure 5

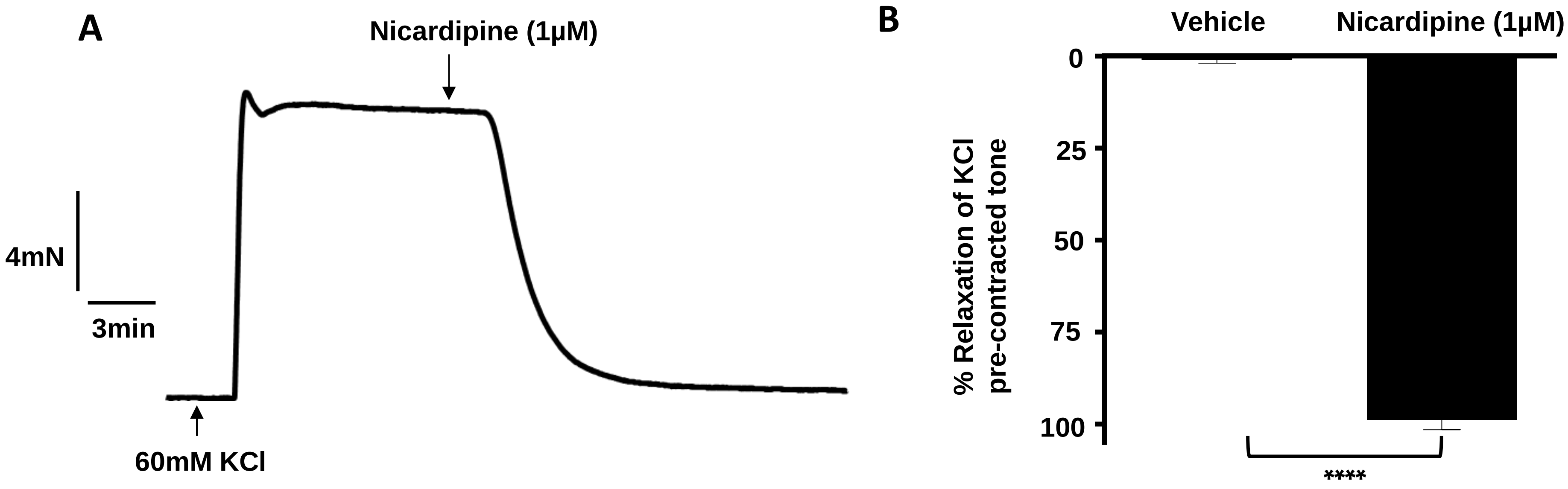

Supplement: Supplementary file 1 — Figure S1. Effect of [Ca2 +]o on U46619 pre-contracted tone of rabbit mesenteric arteries. (A) Representative traces showing the effect of [Ca2 +]o on pre-contracted tone induced by 300nM U46619 on endothelium-intact (Left trace) and endothelium-removed vessels (Right trace). (B) Mean data showing the effect of [Ca2 +]o on U46619-induced pre-contracted vessels. Each point is from n = 4 animals, with at least n = 3 vessel segments per animal. **p < 0.01, ***p < 0.001. Figure S2 Effect of Calhex-231 on CCh-mediated relaxation of pre-contracted tone. Pre-treatment with 3 μM Calhex-231 had no effect on CCh-induced concentration-dependent relaxations of pre-contracted tone. All points are from n = 4 animals, with at least n = 3 segments per animal. Figure S3 Effect of KT5823 on pre-contracted tone. (A) Representative images and (B) mean data showing that 1 μM KT5823 induced about a 25% relaxation of pre-contracted tone which was inhibited by removal of a functional endothelium and co-application of 100 nM CbTx and 100 nM apamin but not by application of 300 μM L-NAME. All points are from n = 3 animals, with at least n = 3 segments per animal. * p < 0.05. Figure S4. Effect of CCh on NO production in isolated ECs measured using DAF-FM fluorescence. (A) Mean data showing that CCh increased basal fluorescence which was inhibited pre-treating cells with 300 μM L-NAME but not by (B) 3 μM Calhex-231. n = at least 50 cells from n = 4 animals. **p < 0.01, ***p < 0.001. Figure S5. Effect of NiCardipine on KCl-precontracted tone. (A) Representative trace and (B) Bar graph of mean data showing that 1 μM NiCardipine (voltage-gated calcium channel inhibitor) completely abolishes 60 mM KCl-induced pre-contracted tone. Bars and error bars are mean values ± s.e.m. from n = 4 animals, with at least n = 3 vessel segments from each animal. ****p < 0.001. [file mmc1.pdf]
